# Letter to the Editor Regarding “Devastating ‘DANA’ Floods in Valencia: Insights on Resilience, Challenges, and Strategies Addressing Future Disasters”

**DOI:** 10.3389/phrs.2025.1609115

**Published:** 2025-10-28

**Authors:** María García-García, Alicia Castro-Fernández, Amparo Ortega-Yago, Alessandro Thione, Víctor García-Bustos

**Affiliations:** ^1^ Plastic Surgery and Burns, Hospital Universitari i Politecnic La Fe, Valencia, Spain; ^2^ Orthopedics and Traumatology, Hospital Universitari i Politecnic La Fe, Valencia, Spain; ^3^ Infectious Diseases, Hospital Universitari i Politecnic La Fe, Valencia, Spain; ^4^ Severe Infection Research Group, Health Research Institute La Fe, Valencia, Spain

**Keywords:** disaster, disaster medicine, floods, epidemiology, natural disasters

Dear Editors,

We read with great interest the work from Martín-Moreno et al. [1] We would like to congratulate the authors for providing an excellent overview of resilience, challenges and strategies addressing future disasters. We would like to contribute a complementary perspective focused on the management of extremity wounds in a disaster setting.

Following the DANA disaster, our Emergency Department (ED) treated more than a hundred patients with extremity wounds related to the flood, providing us an opportunity to analyze injury patterns, microbiology and outcomes. Most patients were men (70%), with a mean age of 47 years. Two-thirds of wounds (68.5%) occurred during cleanup efforts, highlighting the risks faced not only during the disaster but also in its aftermath. Lower extremity wounds predominated (53.7%). While most injuries were superficial abrasions or lacerations, 11% of patients required surgery.

Among hospitalized cases, wound cultures revealed a predominance of Gram-negative organisms (67.7%), particularly *Proteus mirabilis*, *Pseudomonas aeruginosa*, *Escherichia coli*, and *Aeromonas* spp. Extended-spectrum β-lactamase (ESBL) resistance was frequent in *E. coli*, and methicillin-resistant *Staphylococcus aureus* was also detected. Polymicrobial infections occurred in 40% of positive cultures, highlighting the microbiological complexity of disaster-related wounds [2–4].

Our standardized protocol was based on five pillars:

- Wound care: low-pressure, high-volume irrigation with gravity-fed saline (5–12 L; sterile or potable water if unavailable), with selective direct closure.
- Tetanus prophylaxis: toxoid (devitalized tissue, stagnant water exposure) or immunoglobulin (major soft tissue loss, open fractures) administration.
- Antibiotic prophylaxis: in the ED, intravenous Amoxicillin–Clavulanate or Cefazolin; and intravenous Piperacillin-Tazobactam at hospitalization.
- Surgical management: debridement and delayed wound closure in contaminated or infected wounds.
- Multidisciplinary team: coordination between the Emergency Department, Orthopedics, Infectious Disease, Plastic Surgery, Rehabilitation, and Psychology proved essential in minimizing complications and optimizing functional recovery.

In conclusion, our experience illustrates that a structured, evidence-based protocol for wound irrigation, antibiotic use, and surgical management, combined with multidisciplinary coordination, is feasible in disaster contexts. We believe that sharing these practical lessons can support preparedness and improve healthcare responses in future mass-casualty scenarios.

## References

[1] Martin-Moreno JM Garcia-Lopez E Guerrero-Fernandez M Alfonso-Sanchez JL Barach P. Devastating “DANA” Floods in Valencia: Insights on Resilience, Challenges, and Strategies Addressing Future Disasters. Public Health Rev (2025) 46:1608297. 10.3389/phrs.2025.1608297 40357389 PMC12066247

[2] Kespechara K Koysombat T Pakamol S Phoungchit P Panyaphul W. Infecting Organisms in Victims from the Tsunami Disaster: Experiences from Bangkok Phuket Hospital, Thailand. Int J Disaster Med (2005) 3(1–4):66–70. 10.1080/15031430600694244

[3] Edsander-Nord Å. Wound Complications from the Tsunami Disaster: A Reminder of Indications for Delayed Closure. Eur J Trauma Emerg Surg (2008) 34(5):457–64. 10.1007/s00068-008-8802-5 26815990

[4] Wuthisuthimethawee P Lindquist SJ Sandler N Clavisi O Korin S Watters D Wound Management in Disaster Settings. World J Surg (2015) 39(4):842–53. 10.1007/s00268-014-2663-3 25085100 PMC4356884

